# Interactions between Bacteria and Bile Salts in the Gastrointestinal and Hepatobiliary Tracts

**DOI:** 10.3389/fmed.2017.00163

**Published:** 2017-10-03

**Authors:** Verónica Urdaneta, Josep Casadesús

**Affiliations:** ^1^Departamento de Genética, Facultad de Biología, Universidad de Sevilla, Sevilla, Spain

**Keywords:** bile salts, gall bladder, intestinal microbiome, gene regulation, resistance to bile, *Salmonella*, chronic infection

## Abstract

Bile salts and bacteria have intricate relationships. The composition of the intestinal pool of bile salts is shaped by bacterial metabolism. In turn, bile salts play a role in intestinal homeostasis by controlling the size and the composition of the intestinal microbiota. As a consequence, alteration of the microbiome–bile salt homeostasis can play a role in hepatic and gastrointestinal pathological conditions. Intestinal bacteria use bile salts as environmental signals and in certain cases as nutrients and electron acceptors. However, bile salts are antibacterial compounds that disrupt bacterial membranes, denature proteins, chelate iron and calcium, cause oxidative damage to DNA, and control the expression of eukaryotic genes involved in host defense and immunity. Bacterial species adapted to the mammalian gut are able to endure the antibacterial activities of bile salts by multiple physiological adjustments that include remodeling of the cell envelope and activation of efflux systems and stress responses. Resistance to bile salts permits that certain bile-resistant pathogens can colonize the hepatobiliary tract, and an outstanding example is the chronic infection of the gall bladder by *Salmonella enterica*. A better understanding of the interactions between bacteria and bile salts may inspire novel therapeutic strategies for gastrointestinal and hepatobiliary diseases that involve microbiome alteration, as well as novel schemes against bacterial infections.

## Contribution of Bacterial Metabolism to the Intestinal Pool of Bile Salts

Bile salts are produced in the liver from cholesterol, specifically in pericentral hepatocytes, and their daily production is approximately 350 mg (1). Seventeen enzymes convert cholesterol into bile acids, which are transformed into bile salts by the association with Na^+^ or K^+^ ions. There are four types of bile salts: primary and secondary, conjugated, and non-conjugated (2, 3). Conjugation involves the formation of an amide bond with either taurine or glycine.

Primary bile salts are the immediate products of cholesterol degradation. Variations in the pool of primary bile salts occur among vertebrates: for instance, in humans and rats, the primary bile salts are cholate and chenodeoxycholate while in mice they are cholate and muricholate (2). Intestinal bacteria transform primary bile salts into secondary bile salts by removal of the hydroxyl group at C7. In humans, secondary bile salts are deoxycholate (DOC, from cholate) and litocholate (from chenodeoxycholate) (4, 5). The hydroxyl groups of bile salts protrude in the same direction, a feature that is partly responsible for their detergent activity because it confers high-water solubility and generates delimited hydrophilic and hydrophobic regions (6).

Intestinal anaerobes play a major role in bile salt metabolism (7, 8), and the main transformations are as follows:

(i) Hydrolysis of the amide bond between the glycine or the taurine conjugated to the steroid nucleus. This reaction, called deconjugation, makes bile salts available as substrates for further modifications by the intestinal microbiota and is, therefore, crucial in bile biotransformation (9–11). Deconjugation is catalyzed by bacterial enzymes known as bile salt hydrolases (BSH), which are widespread in the bacterial world and include Gram-positive intestinal species like *Lactobacillus* (12–16), *Enteroccocus* (17, 18), *Bifidobacterium* (19–21), and *Clostridium* (22). BSH activity has also been reported in the commensal, Gram-negative *Bacteroides* spp. (23), and in the Archaea domain, specifically in species of the intestinal microbiome, such as *Methanobrevibacter smithii* and *Methanosphera stadmanae* (23). The high levels of identity found between BSH of different domains suggest horizontal gene transfer (23). Additionally, BSH genes show high redundancy in the gut ecosystem, and the number of BSH paralogs varies from strain to strain; for instance, in some serovars of *Lactobacillus plantarum* four different functional BSH genes have been described (13, 24). A potential selective value of BSH activity is enhancement of bile tolerance, thus facilitating survival in the gut (23, 25). Furthermore, bile salts serve as acceptors of electrons generated by fermentation while glycine and taurine can be used as sources of carbon and nitrogen (6).
(ii) 7α/β-dehydroxylation converts primary bile salts into secondary bile salts. Examples are 7α-dehydroxylation of cholate and chenodeoxycholate yielding deoxycholate and lithocholate, respectively, and 7β-dehydroxylation of ursodeoxycholate yielding lithocholate (11). These biotransformations occur in the human colon, and deoxycholate and lithocholate are the predominant bile salts in human feces (7, 26). A pre-requisite for these transformations is deconjugation because 7α/β-dehydroxylation occurs in free bile salts (27). Unlike BSH activity, only a small number of bacterial species belonging to the class Clostridia have 7α/β-dehydroxylation activity (28). Transformation of primary into secondary bile salts requires transport of free primary bile salts into the bacterial cell, which is carried out by the proton-dependent bile acid transporter BaiG (29). Once inside the cell, a series of reactions occur, beginning with ligation of the bile salt to CoA in a Mg^2+^- and ATP-dependent reaction catalyzed by CoA ligase (30). The bile salt-Coa thioester is then oxidized at the 3-hydroxy group by a 3α-hydroxysteroid dehydrogenase (31). After oxidation, NAD-dependent flavoproteins synthesize 3-dehydro-Δ^4^-chenodeoxycholate or 3-dehydro-Δ^4^-cholate and 3-dehydro-Δ^4^-ursodeoxycholate, respectively (31, 32), making the bile salt chemically labile for irreversible 7α-dehydration (7). The enzymes involved in further steps of this pathway have not yet been identified; they may include oxidoreductases that catalyze reduction to secondary bile salts (7). A potential advantage for 7α/β-dehydroxylating bacteria might be favorable niche competition upon exclusion of microorganisms sensitive to secondary bile salts (7); additionally, production of reduced NADPH might be energetically useful by providing proton motive force (9).
(iii) Numerous enteric especies (e.g., *Clostridium, Peptostreptococcus, Bacteroides, Eubacterium*, and *Escherichia coli*) can perform oxidation and epimerization of hydroxy groups at the positions C3, C7, and C12 of bile salts, generating isobile (β-hidroxy) salts. Examples are 3-oxocholanoate and isocholate; 7-oxocholanoate and 7-epicholate; and 12-oxocholanoate and 12-epicholate (11). Oxidation and epimerization are catalyzed by hydroxysteroid dehydrogenase. Epimerization is a reversible stereochemical change from α to β configuration or *vice versa*, with the formation of a stable oxo-bile salt intermediate (7). These modified bile salts (epimers and isobile salts) are usually recycled to the liver and repaired before rejoining bile (9).

Some of these biotransformations contribute to the salvage of bile salts that escape active transport in the distal ileum during enterohepatic circulation. Particularly, deconjugation and 7α-dehydroxylation increases hydrophobicity and Pk_a_ of bile salts, facilitating their recovery by passive transport in the colon epithelium (7).

In the absence of microbial transformations, the diversity of the bile salt pool decreases (33). The intestinal microbiota has an active role in the regulation of bile salt synthesis: bacterial metabolism decreases the level of taurine-conjugated muricholic acid, a farnesoid X receptor (FXR) antagonist that inhibits FXR signaling in the intestine. FXR signaling reduces the expression of cholesterol 7α-hydrolase (CYP7a1), a rate-limiting enzyme in bile acid synthesis; and as a consequence, primary bile acid synthesis is reduced. Hence, the gut microbiota regulates not only secondary bile salt metabolism but also primary bile salt synthesis by alleviating FXR inhibition (34).

**Figure 1:**
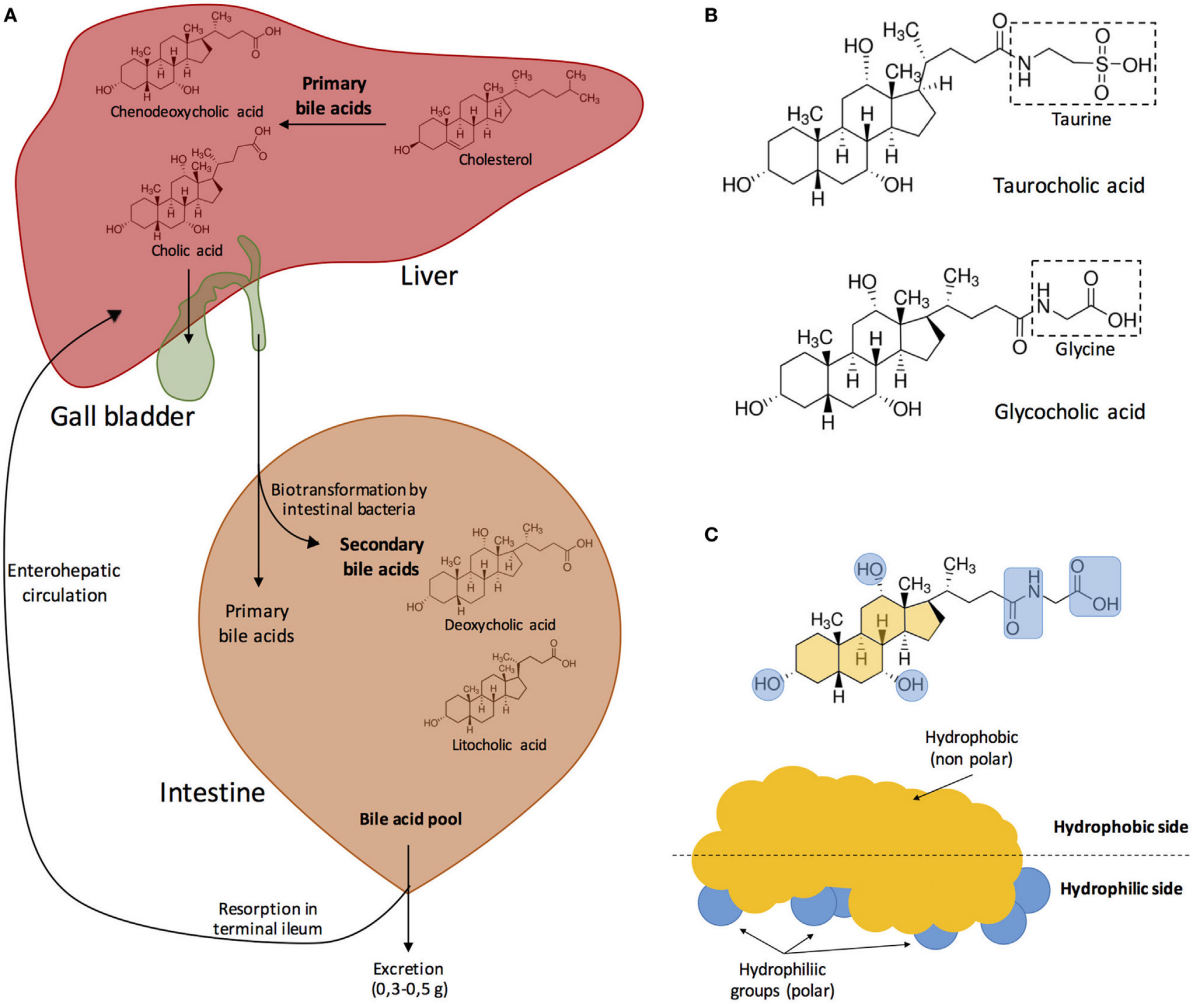
**(A)** Synthesis of the major bile acids of human bile and circulation in the hepatic, biliary, and digestive systems. Bile acids are transformed into bile salts by the association with Na^+^ or K^+^ ions. Primary bile acids are stored in the gall bladder. After food ingestion, bile released into the small intestine contains 5–15 g of bile acids. In the intestine, bile acids are modified by the effect of intestinal microbiota. Both primary and secondary bile acids are reabsorbed in the terminal ileum into the blood stream. Modified from Fontana et al. (3). **(B)** Bile acids can be conjugated with either glycine or taurine. In this example, cholic acid becomes either taurocholic acid or glycocholic acid after conjugation with taurine or glycine, respectively. **(C)** Bile acids are amphipathic, and their hydrophobic side associates with the surface of lipid droplets while the polar groups interact with water creating a stable emulsion of small, mixed micelles. Modified from Hofmann (5) and Vander et al. (35).

Figure 1 depicts the process of synthesis of the most abundant bile salts present in human bile and their circulation trough the hepatic, biliary, and digestive systems, as well as their chemical structure and physicochemical properties.

## Modulation of the Gut Microbiome by Bile Salts

The human body is a complex ecosystem where the number of commensal bacterial cells are roughly as abundant as “human” cells (36). Particularly, the gut microbiome has one of the highest bacterial densities in nature (10^12^ bacteria/g feces, wet weight) (7, 37), and may be viewed as a dynamic community that has co-evolved with their host to facilitate digestion and absorption of complex food components (38). In this symbiotic process, the host must control bacterial colonization of the small intestine since bacterial overgrowth can lead to deficient absorption of nutrients due to bacterial competition with the host.

The factors that can induce changes in the gut microbiome include transit time, abundance of proteolytic enzymes, antimicrobial peptides, diet, age, antibiotic use, disease, and bile (7, 39, 40). Several studies have reported the effect of bile salts on gut microbial communities. According to Kakiyama et al. (40), there is a connection between liver health, bile composition, and gut microbiome structure: as an example, patients with cirrhosis exhibit lower levels of fecal bile salts, which reflects a drop in conversion of primary to secondary bile salts. This decrease may be correlated with an alteration of the gut microbiome upon overgrowth of enteric bacteria (potential pathogens) and decreased abundance of 7α-dehydroxylating Gram-positives like *Lachnospiraceae, Ruminococcaceae*, and *Blautia*. On the other hand, the amount of bile released into the intestine can alter gut colonization: a low level of bile salts favors proliferation of Gram-negative bacteria (including pathogens), while high levels of bile salts favors the proliferation of Gram-positive bacteria and reduction of the Gram-negative *Bacteroides* (41).

High-fat diets, characteristic of Western populations, also affect the structure of the intestinal microbiome by altering the bile salt pool (42, 43). Experiments carried out with an animal model for inflammatory bowel disease (interleukin-10-deficient mice), have shown that the gut microbiome community is altered when the animals are fed with a high saturated fat diet. In particular, the authors detected proliferation of the sulfite-reducing pathobiont *Bilophila wadswortia* (42), a bacterial species that is difficult to detect in healthy organisms but is associated with appendicitis and intestinal inflammation (44, 45). A diet high in saturated fat favors taurine conjugation of bile salts over glycine conjugation, increasing organic sulfur availability for *B. wadswortia* (43).

Bile participates in maintaining the intestinal homeostasis as an activator of the FXR and of the Vitamin D nuclear receptor (VDR) (45–48). FXR is a transcription factor from the nuclear receptor family and functions as the main sensor of intracellular bile levels. It is most abundant in tissues exposed to bile salts like the liver and the intestine (49, 50) and modulates intestinal innate immunity (49). The role of FXR in antibacterial defense was inferred from the observation that mice with obstructed bile ducts suffered from microbial proliferation followed by invasion of the epithelium; these symptoms were alleviated by either a synthetic FXR ligand or by bile salts (46, 47, 51). FXR activates genes involved in enteric protection and inhibition of bacterial overgrowth like the angiogenin gene *Ang1* and the nitric oxide synthase gene *iNos* (46, 50). Furthermore, bile salts can induce the synthesis of cathelicidin in biliary epithelial cells. Cathelicidins are antimicrobial peptides that prevent bacterial infections *in vivo* (51) including those caused by pathogenic *E. coli* (52) and are involved in innate immunity (53). Bile salts induce the extracellular protein kinase (ERK 1/2) pathway which in turn activates the VDR receptor, resulting in cathelicidin synthesis (48).

## Alteration of the Microbiome-Bile Salt Homeostasis in Hepatic and Intestinal Diseases

Because bile salts control the structure of the intestinal microbiome and the microbiome regulates the composition and size of the bile salt pool, alteration of the microbiome–bile salt homeostasis can have multiple pathological consequences. In cirrhotic patients, for instance, a shrinking bile salt pool may alter the intestinal microbiome by increasing the size of bacterial populations that produce proinflammatory molecules, which trigger a feedback loop as inflammation downregulates bile acid synthesis in the liver (54, 55). As cirrhosis progresses, decreased concentrations of bile salts in the small intestine permit bacterial overgrowth, which many contribute to cirrhosis complications like intestinal endotoxemia and hepatic encephalopathy (54, 56). Microbiome-induced alteration of the bile salt pool may also play a role in non-alcoholic fatty liver disease by impairing the activity of bile salt receptors and bile salt transporters (57).

Bile salt metabolism and signaling is also impaired in cholestasis, which causes accumulation of bile salts in the liver with concomitant hepatocyte injury and inflammation. Patients with chronic cholestasis may be at higher risk of developing hepatocellular and bile duct cancer (58). This hypothesis is consistent with the observation that bile salts can promote cell proliferation by activating mitogenic pathways in the hepatobiliary tract (59).

The composition of the intestinal microbiota may also play a role in progression of colorectal cancer (58), and alteration of the composition of bile salt pool may be indirectly involved in this condition by favoring intestinal colonization by Firmicutes and Proteobacteria with concomitant decrease of Bacteriodetes (60).

In the stomach, alterations such as diet and drug use may favor the colonization of pathogens like *Helicobacter pylori* (61), which promotes mucosal inflammation of the gastric epithelium and has been identified as the strongest risk factor for gastric adenocarcinoma (62). In the particular case of adenocarcinomas associated with *H. pylori*-related proximal atrophic gastritis, an increase in concentration of bile salts in the distal stomach may prompt migration of the pathogen to the proximal stomach (63).

Other *Helicobacter* species have also been isolated from patients with biliary diseases like cholelithiasis, cholecystitis, gall bladder polyp, and gall bladder and biliary tract cancers (64–66).

## Bile Salts as Environmental Signals for Intestinal Bacteria

The expression of certain bacterial loci is regulated by bile salts, which may serve as signals that identify the intestinal environment. For instance, *Shigella* invasion genes and certain *Salmonella* genes belonging to the PhoPQ regulon, which controls multiple virulence traits, are upregulated in the presence of bile salts (67–69). In contrast, expression of the *Salmonella enterica* pathogenicity island 1 (SPI-1), which encodes a type 3 secretion system necessary for invasion of the ileal epithelium, is inhibited by bile salts (70). This repression may prevent synthesis of invasion proteins in intestinal environments that are not appropriate for invasion. In *Vibrio cholerae*, bile salts activate transcription of genes involved in virulence and biofilm formation (71, 72). Changes in gene expression and/or protein synthesis in the presence of bile have been also described in the Gram-negative *Campylobacter jejuni* (73) and in the Gram-positives *Enterococcus faecalis* (74) and *Listeria monocytogenes* (75).

The molecular mechanisms involved in transcriptional regulation by bile salts are known in certain cases. In *Vibrio* spp., bile salts bind specific receptors in the bacterial surface, activating signal transduction pathways that modulate gene expression patterns. For instance, the inner membrane proteins VtrA and VtrC of *Vibrio parahaemolyticus* are part of a bile salt-sensitive signal transduction system, and binding of bile salts to VtrC activates expression of type 3 secretion genes (76). Inner membrane proteins also control virulence gene expression in *V. cholerae* in response to bile salts: the TcpP/TcpH and ToxR/ToxS pairs constitute bile salt-sensitive signal transduction systems that control transcription of *toxT* (77, 78). In turn, ToxR is a transcriptional activator of genes encoding the cholera toxin and the toxin co-regulated pilus (79). In *S. enterica*, repression of invasion in the presence of bile salts involves postranscriptional destabilization of the transcription factor HilD (80).

Bile salts also control pathogenesis of *Clostridium difficile*, a Gram-positive spore-forming anaerobe (81). *C. difficile* vegetative cells produce enterotoxins (e.g., TccA and TcdB) that cause inflammation and diarrhea. This process is controlled by the *cspBAC* locus (82). Taurocholate, a conjugated primary bile salt, activates germination of the spores through interaction with the spore receptor CspC (83). Interestingly, the secondary bile salt deoxycholate can also promote spore germination but inhibits vegetative growth (84). On the other hand, chenodeoxycholate, an unconjugated primary bile salt, inhibits spore germination and is a competitive inhibitor of taurocholate (85). Mice treated with broad spectrum antibiotics show increased susceptibility to *C. difficile* infection if their pools of secondary bile salts are diminished (86). These observations suggest that the type of bile salt predominant in the medium serves as an environmental signal to either remain dormant or trigger spore germination.

In enterohemorrhagic *E. coli* O157:H7, bile reduces the expression of genes of the enterocyte effacement (LEE) pathogenicity island (87). When the concentration of bile decreases in downstream regions of the intestine, the LEE locus is activated. LEE expression induces attaching and effacing pathogenesis, and ultimately results in loss of the intestinal epithelial barrier (88). This pathology, which occurs specifically in the large intestine, provides another example of bile salt-mediated intestinal signaling (87).

## Bile Salts as Antimicrobial Agents

Bile inhibits bacterial growth (69). In patients with primary cirrhosis, where biliary tract sterility is disrupted (89), the administration of bile salts decreases endotoxin accumulation in biliary epithelial cells (90). The inhibitory effect of bile salts on bacterial growth can be also observed under laboratory conditions, and appears to be the consequence of multiple injuries caused by salts to the bacterial cell.

### Disruption of Bacterial Cell Membranes

Bile acids are surface active, amphipathic molecules, and their detergent activity damages cell membranes. Not surprisingly, many bile-sensitive mutants of both Gram-negative and Gram-positive bacteria carry mutations that impair membrane integrity. Likewise, electron microscopy studies have described a shrunken phenotype in *Propionibacterium freudenreichii* cells exposed to bile (91). Enzymatic assays in *E. coli, Clostridium perfringens*, and *Lactobacillus acidophilus* have shown that bile causes leakage of intracellular material (92, 93). Factors that influence the severity of membrane disruption are as follows:

(i) Concentration of bile, high concentrations will dissolve membrane lipids, causing leakage and cell death (94). Low concentrations of bile may have more subtle effects on membrane fluidity and permeability by altering membrane-bound proteins or increasing trans-membrane divalent cation flux. Low levels of bile can also alter the hydrophobicity of the cell surface (92, 93, 95, 96).
(ii) Type and structure of bile salts. Bile salts conjugated with taurine or glycine are fully ionized at physiological pH and for this reason they remain in the outer hemi-leaflet of the membrane; on the contrary, unconjugated bile salts passively cross membranes and enter the cell (97–99).
(iii) Membrane architecture and composition. Changes in lipopolysaccharide (LPS), membrane electric charge, hydrophobicity, lipid fluidity, and fatty acid composition alter bile resistance levels in multiple bacterial species (e.g., *E. coli, L. monocytogenes*, and *L. acidophilus*) (100–103).

### Induction of Macromolecular Instability: DNA Damage

Upon entry into the bacterial cell, bile salts cause nucleic acid damage. In *E. coli*, sodium chenodeoxycholate and sodium deoxycholate activate the SOS response (104). Increased transcription of the SoxRS regulon genes *osmY* and *micF* suggests that DNA oxidative damage may occur upon exposure to bile salts (105, 106). In *S. enterica*, bile increases the frequency of nucleotide substitutions, frameshifts, and chromosomal rearrangements (107), and the mutational spectrum of bile suggests that one primary lesion may be oxidative damage of cytosine (108). Bile salts also induce curing of the *Salmonella* virulence plasmid (109), a feature common among DNA damaging agents (110).

### Misfolding and/or Denaturation of Proteins

The detergent activity of bile salts can alter the conformation of proteins. Not surprisingly, synthesis of chaperones DnaKJ and GroESL (91, 111), which assist in proper folding of proteins, increases in the presence of bile salts.

### Chelation of Iron and Calcium

Bile salts are able to chelate iron and calcium. In the case of iron, the primary bile acids taurocholic and glycocholic can form soluble Fe^2+^–bile salt complexes. This binding increases intestinal iron uptake (112). Because both the host and the microbiota require iron for fundamental cellular processes, bile salts may withhold iron from microorganisms, limiting their growth (113).

Calcium ions (Ca^2+^) can bind to micelles of bile salts conjugated with either taurine or glycine. This binding reduces Ca^+2^ precipitation, thereby decreasing the contribution of calcium to the formation of gallstones (114). Calcium is also a signal involved in motility, cell cycle and cell division, control of gene expression, and chemotaxis (115, 116). Hence, shortage of Ca^2+^ upon bile salt chelation may also limit bacterial proliferation.

## Resistance to Bile in Enteric Bacteria

The ambivalent nature of bile salts as signals of the host environment and as antibacterial agents requires that intestinal bacteria can cope with bile-induced injuries. Not surprisingly, bacterial species adapted to the mammalian intestine are resistant to bile salts, a trait exploited for the design of selective microbiological media such as the MacConkey agar used in the identification of genera of the family Enterobacteriaceae.

Addition of ox bile or individual bile salts to microbiological media is also a strategy to study resistance to bile salts under laboratory conditions. Using this reductionist approach, genetic and biochemical analyses have identified cell functions and mechanisms involved in bile resistance in a number of species including the model organisms *E. coli* and *S. enterica* (25, 117, 118) (Table 1). An overall conclusion from these studies is that resistance to bile involves multiple cell functions and mechanisms.

**Table 1:** Genetic loci that contribute to bile resistance in enteric bacteria.

Gene	Function of encoded protein(s)	Phenotype of mutants	Reference
*phoPQ*	Two-component system	Bile sensitive	(69, 119)
*marRAB*	Regulatory genes	Bile sensitive	(120)
*acrAB*	Efflux pump	Bile sensitive	(119–121)
*tolQRA, tolC*	Cell envelope	Bile sensitive	(119, 122)
*dam*	DNA adenine methylase	Bile sensitive	(107, 119, 123)
*wecD, wecA*	Biosynthesis and assembly of enterobacterial common antigen	Bile sensitive	(124)
*xthA* and *nfo*	Exonuclease and endonuclease, respectively, involved in DNA repair	Bile sensitive	(108)
*recA, B, C, D, J*	Repair and maintenance of DNA	Bile sensitive	(108)
*dinB*	DNA repair	Bile sensitive	(108)
*seqA*	GATC-binding protein	Bile sensitive	(119, 125)
*hupA*	DNA-binding protein	Bile sensitive	(119)
*mrcA, mrcB*	Penicillin-binding proteins 1A and 1B	Bile sensitive	(119)
*sanA*	Uncharacterized membrane protein	Bile sensitive	(119)
*sbcB*	Exonuclease, involved in DNA repair	No phenotype, locus upregulated by bile	(108)
*yciF*	Unknown function	No phenotype, locus upregulated by bile	(126)
STM4242	Unknown function	No phenotype, locus upregulated by bile	(126)
*rpoS*	General stress response	Bile sensitive, locus upregulated by bile	(127)
*prc*	Peptidoglycan remodeling	Bile-hyperesistant	(128)
*rfa*	Lipopolysaccharide synthesis	Bile-hyperesistant	(117, 129)
*toxR, toxT*	Regulatory genes	Bile sensitive	(130)

### Bacterial Cell Envelope

The cell envelope of Gram-negative bacteria contains three layers: the cytoplasmic (inner) membrane, the peptidoglycan cell wall, and the outer membrane. The outer membrane is asymmetrical: its inner leaflet consists mainly of phospholipids while the outer leaflet is almost entirely composed of a glycolipid known as LPS (131). Loss of the O-antigen in the LPS results in decreased resistance to bile (117, 129); on the contrary, very long O-antigen chains increase bile resistance (132). The relevance of the LPS structure in bile resistance is further supported by the observation that *S. enterica* mutants hyper-resistant to bile often carry mutations in LPS transport genes (127).

Another cell envelope component that contributes to bile resistance is the enterobacterial common antigen (ECA), a family-specific glycolipid present in the outer leaflet of the outer membrane. In *S. enterica*, mutations in genes involved in ECA synthesis cause bile sensitivity (124).

Bile salts also induce peptidoglycan remodeling, and remodeling increases bile resistance. Growth of *S. enterica* in the presence of a sublethal concentration of DOC is accompanied by a reduction in the amount of Braun lipoprotein (Lpp) anchored to peptidoglycan (133). Because Lpp-containing muropeptides provide covalent linkage between the outer membrane and the peptidoglycan layer, reduction of this union may increase flexibility in the cell envelope, perhaps altering outer-membrane fluidity. Growth of *S. enterica* in the presence of DOC is also associated with a decrease in 3–3 crosslinks between the sugar components of peptidoglycan (*N*-acetylmuramic acid and *N*-acetylglucosamine), suggesting that low crosslinking may increase bile resistance (133).

### Efflux Pumps

Even though the bacterial envelope provides a barrier that reduces bile salt uptake, bile salts can enter the cell by diffusion or by passage through porins like OmpF. As a consequence, active efflux is necessary to reduce their concentration inside the cell (134). Among the efflux systems found in enterobacterial species, AcrAB–TolC is the best characterized (135–138). It comprises the outer-membrane protein channel TolC, the proton force-dependent transporter AcrB located in the inner membrane, and the periplasmic protein AcrA, which aids in efflux by bridging the TolC and AcrA integral membrane proteins (139). The AcrAB–TolC efflux pump is able to transport a diverse array of compounds with little chemical similarity (140), and is essential for bile resistance (120, 121, 141, 142). The genes encoding the AcrAB–TolC multidrug efflux system are under the control of a transcriptional regulator known as RamA. In turn, transcription of the *ramA* gene is activated by bile salts, mainly by relieving transcriptional repression exerted by the RamR protein (138, 143).

### DNA Repair Mechanisms

DNA adenine methylase (Dam^–^) mutants of *S. enterica* are bile sensitive (123, 144), and genetic analysis unveils the involvement of Dam-directed mismatch repair (107): mutations in any of the mismatch repair genes *mutH, mutL*, or *mutS* suppress bile sensitivity in *dam* mutants, providing evidence that bile sensitivity is caused by MutHLS activity. *Salmonella* MutHLS^−^ mutants are not sensitive to bile, indicating that bile-induced DNA damage can be repaired by mechanisms other than Dam-dependent mismatch repair. In Dam^−^ mutants, however, lack of DNA strand discrimination causes DNA strand breakage when the MutHLS systems deal with bile-induced lesions (107).

Surveys of bile sensitivity among *S. enterica* DNA repair mutants have revealed that base excision repair (BER), SOS-associated DNA repair, and recombinational repair by the RecBCD enzyme are required to cope with bile-induced DNA damage (108). In contrast, nucleotide excision repair (NER) is dispensable. The observation that bile resistance requires BER but not NER suggests that bile-induced DNA lesions are unlikely to be bulky, thus providing indirect support for the occurrence of oxidative damage (107, 108).

Several lines of evidence suggest that bile salts may impair DNA replication in *Salmonella*: (i) *dinB* mutations confer bile sensitivity, suggesting that SOS-associated translesion DNA synthesis may be required to overcome bile-induced DNA damage; (ii) RecB^–^, RecC^–^, and RecA^−^ RecD^−^ double mutants are also bile sensitive, indicating that survival to bile may require RecB-dependent homologous recombination (108). A tentative scenario is that primary lesions (e.g., oxidized cytosine moieties) may trigger BER. As a consequence, BER exonucleases will produce DNA strand breaks as an intermediate step in the DNA repair process. Furthermore, as indicated above, DNA strand breaks can also be formed as a consequence of MutHLS activity. These DNA strand breaks may impair progression of DNA replication forks, inducing the SOS response (see below); as a consequence, DinB-mediated translesion DNA synthesis may occur (108). It is also conceivable that bile-induced lesions could directly block DNA replication, thus inducing the SOS response in a direct fashion. In such a scenario, the need of homologous recombination mediated by the RecBCD enzyme complex might reflect the occurrence of stalled DNA replication forks (108).

### Stress Responses

Given the multiple injuries caused by bile salts to the bacterial cell, it is not surprising that stress regulons are induced in the presence of bile. The relevance of the DNA damage responsive SOS regulon, mentioned already in the previous section, is supported by the observation that LexA(Ind^–^) mutants, which are unable to induce the SOS response, are bile sensitive (108). Somehow surprisingly, the oxidative damage-responsive OxyR and SoxRS regulons, which are also activated by bile salts, are not essential for bile resistance (108). A tentative explanation is that redundance may exist in the stress responses of the bacterial cell so that certain functions can be backed up by others.

The RpoS-dependent general stress response is essential for bile resistance, and RpoS^−^ mutants are bile sensitive. In *S. enterica*, an interesting feature of the RpoS response is inhibition of conjugation in the presence of bile, which involves posttranscriptional control of *rpoS* mRNA and *ricI* mRNAs by the small regulatory RNA RprA (145). The *ricI* gene encodes a cytoplasmic membrane protein that inhibits plasmid transfer by direct interaction with the conjugation apparatus protein TraV. RpoS^−^ mutants of *S. enterica* are avirulent in the mouse model of typhoid (146, 147), and it seems reasonable to hypothesize that one cause of avirulence may be bile sensitivity.

Transcriptomic analysis has identified additional, RpoS-independent stress-inducible genes that increase their expression in the presence of DOC (*cspD, uspA, aphC*, etc.) (127). Although the contribution of these loci to bile resistance remains to be established, the number and variety of stress functions activated by bile salts supports the view that multiple stress responses contribute to bile resistance. Some such responses appear to be essential while others are not.

## Colonization of the Hepatobiliary Tract by *Salmonella enterica*

*Salmonella* stands out among bile-resistant bacterial genera because of its ability to colonize the hepatobiliary tract causing chronic infection. The current taxonomy defines six subspecies of *S. enterica*, which are in turn classified into serovars. The majority of serovars belong to subspecies *enterica* (148), which colonizes warm-blooded vertebrates (149) and accounts for 99% of human infections by *Salmonella* (150). Serovars belonging to subsp. *enterica* differ in host specificity and in the type of disease they produce. Some serovars are host-restricted, while others can infect a wide variety of animal hosts (151).

**Figure 2:**
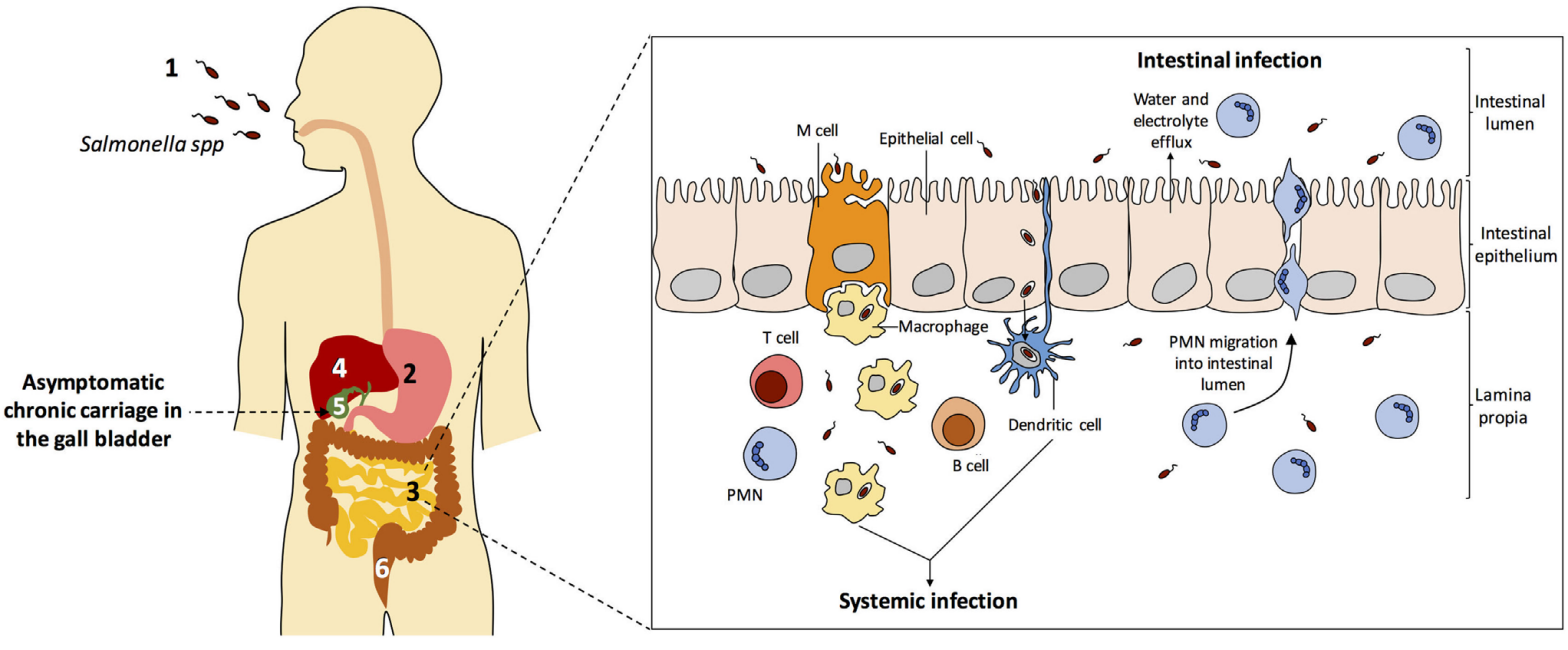
*Salmonella enterica* infection pathogenesis. (1) *Salmonella* infection starts with the ingestion of contaminated food or water. (2) Salmonellae invade intestinal epithelial cells and migrate to the *lamina propria*. (3) Two types of infection can occur: gastroenteritis and systemic infection. (4) During systemic dissemination, *Salmonella* colonizes the liver, the spleen, and the bone marrow. (5) From the liver, *Salmonella* reaches the gall bladder and can cause chronic carriage. (6) *Salmonella* carriers shed bacteria into the small intestine upon secretion of bile, and feces contain *Salmonella* cells. Inspired from Ref. (154–157).

Figure 2 depicts the biology of *Salmonella* infections in humans. All diseases start upon invasion of the intestinal epithelium, often through M cells. Translocation across the intestinal epithelium is mediated by the virulence-associated type 3 secretion system encoded by *Salmonella* pathogenicity island 1 (SPI-1) (152), and invasion allows the bacteria to reach lymphocytes B and T below Peyer patches (153). Once the epithelium is crossed, *S. enterica* can produce three main types of infection: gastroenteritis, systemic infection, and asymptomatic chronic carriage.

(i) Gastroenteritis, a self-limited infection of the terminal ileum and colon which is the most common *Salmonella* infection worldwide, with more that 90 million cases per year (158). Gastroenteritis is produced by typhoidal serovars, especially Typhimuriun and Enteritidis. A localized inflammatory response induces infiltration of polymorphonuclear leukocytes, which helps to contain bacterial dissemination beyond the intestinal submucosa. Secretion of fluids and electrolytes in the small and large intestines produces diarrhea.
(ii) Systemic infection is produced by *Salmonella* serotypes that invade intestinal macrophages and disseminate inside the organism trough the lymphatic system, permitting colonization of internal organs such as the liver, the spleen, the bone marrow, and the gall bladder (Figure 2). In humans, typhoid and paratyphoid fever are caused by serotypes Typhi and Paratyphi, respectively. These infections are associated with high morbidity and mortality rates (159). Typhoid fever is estimated to cause over 20 million illnesses and over 200 thousand deaths worldwide, while the number of cases of paratyphoid fever is estimated over 5 million (160).
(iii) Chronic carriage. A fraction of individuals recovering from typhoid fever become asymptomatic, life-long carriers of *S*. Typhi. Non-typhoidal *Salmonella* serovars can also cause persistent infections, either associated with cholecystitis or asymptomatic, although the duration of carriage is usually limited to several months. The mesenteric lymph nodes, the liver, and the gall bladder appear to be the main *Salmonella* reservoirs during chronic carriage (161).

Colonization of the gall bladder by *Salmonella* in asymptomatic carriers permits shedding of *Salmonella* cells into the intestine upon gall bladder contraction, with concomitant release of bile. From the small intestine, the bacteria travel downwards in the gut, ultimately being released with feces (153, 155). Aside from its epidemiological implications, chronic *Salmonella* carriage may predispose to gall bladder cancer, mainly as a consequence of chronic cholecystitis (162–164).

The bacterial lifestyle in the gall bladder is one of the less known aspects of *Salmonella* biology and a fascinating scientific conundrum. How is it possible that the high concentration of bile salts present in the gall bladder permits *Salmonella* survival? How is it possible that *Salmonella* survival in such a harsh environment can last for a lifetime? Answers to these questions are not simple; survival of *Salmonella* in the gall bladder appears to involve several adaptive strategies, which may be perhaps simultaneously adopted by subpopulations as a bet hedging strategy.

(i) A fraction of the *Salmonella* population may escape from bile salts by invasion of the gall bladder epithelium in a SPI-1-dependent manner, followed by replication in a vacuole (165). In this situation, extrusion of infected epithelial cells and release of *Salmonella* cells into the lumen has been observed. According to Gonzalez-Escobedo and Gunn (166), this mechanism could be important in maintaining the chronic carrier state and dissemination, because the bacteria released could either re-infect the epithelium or be shed into the medium.
(ii) Gallstones may play a major role in chronic infection. Using a murine model of typhoid carriage, John Gunn and co-workers have provided evidence that *S*. Typhimurium can form biofilms on the surface of cholesterol gallstones. The biofilm matrix provides high resistance to antimicrobial agents (167, 168), thereby explaining why antibiotic therapy is ineffective in carriers of *S. enterica* serovar Typhi who suffer from gallstone formation (169). This view is supported by the fact that *S. typhi* cells are detected on gallstones from human typhoid carriers (169).
(iii) Planktonic *Salmonella* cells can multiply in the gall bladder lumen, presumably using phospholipids as carbon and energy sources (170). How these unprotected cells endure the bactericidal activities of bile remains unknown. A tentative speculation is that activation of bile-responsive stress responses may generate cell lineages in which resistance to bile is maintained by feedback loops that are transmissible through cell division. In addition, bile-resistant mutants may appear, especially during longtime colonization as bile salts are mutagenic (108).

## Concluding Remarks and Future Perspectives

The composition of the bile salt pool is shaped by bacterial metabolism, and bile salts are used as physiological signals by both bacteria and hepatic cells. This entangled relationship is made even more complex by the fact that bile salts are antibacterial agents. A better understanding of the contribution of the bile salt pool to gastrointestinal and hepatobiliary homeostasis may inspire novel therapeutic strategies for conditions that involve microbiome alteration (e.g., cirrhosis, fatty liver disease, cholestasis, colorectal cancer, and certain types of *Helicobacter*-associated cancer). In turn, knowledge of mechanisms of bile resistance in intestinal pathogens may stimulate novel schemes to combat infectious diseases. For instance, eradication of *Salmonella* Typhi from the gall bladder of chronic carriers by procedures other than cholecystectomy might reduce chronic carriage of typhoid, a public health problem aggravated by global travel.

## Author Contributions

VU: literature search, writing, and drawing of figures. JC: literature search and writing.

## Conflict of Interest Statement

The authors declare that the research was conducted in the absence of any commercial or financial relationships that could be construed as a potential conflict of interest.
